# Heart Failure Is a Risk Factor for Suffering and Dying of *Clostridium difficile* Infection. Results of a 15-Year Nationwide Study in Spain

**DOI:** 10.3390/jcm9030614

**Published:** 2020-02-25

**Authors:** Manuel Méndez-Bailón, Rodrigo Jiménez-García, Valentín Hernández-Barrera, Javier de Miguel-Díez, José M. de Miguel-Yanes, Nuria Muñoz-Rivas, Noel Lorenzo-Villalba, David Carabantes-Alarcon, José J. Zamorano-León, Paloma Astasio-Arbiza, Paloma Ortega-Molina, Ana López-de-Andrés

**Affiliations:** 1 Internal Medicine Department, Clínico San Carlos University Hospital, 28040 Madrid, Spain; manuel.mendez@salud.madrid.org; 2 Medicine Department, Complutense University of Madrid (UCM), 28040 Madrid, Spain; 3 Clínico San Carlos Hospital Biomedical Research Institute (IdISSC), 28040 Madrid, Spain; 4 Department of Public Health & Maternal and Child Health, Faculty of Medicine, Universidad Complutense de Madrid, 28040 Madrid, Spain; dcaraban@ucm.es (D.C.-A.); jjzamorano@ucm.es (J.J.Z.-L.); pastasio@med.ucm.es (P.A.-A.); pomolina@med.ucm.es (P.O.-M.); 5 Preventive Medicine and Public Health Teaching and Research Unit, Health Sciences Faculty, Rey Juan Carlos University, Alcorcón, 28922 Madrid, Spain; valentin.hernandez@urjc.es (V.H.-B.); ana.lopez@urjc.es (A.L.-d.-A.); 6 Respiratory Department, Hospital General Universitario Gregorio Marañón, Facultad de Medicina, Universidad Complutense de Madrid, Instituto de Investigación Sanitaria Gregorio Marañón (IiSGM), 28009 Madrid, Spain; javier.miguel@salud.madrid.org; 7 Internal Medicine Department, Hospital General Universitario Gregorio Marañón, Madrid, Facultad de Medicina, Universidad Complutense de Madrid (UCM), 28009 Madrid, Spain; josemaria.demiguel@salud.madrid.org; 8 Medicine Department, Hospital Universitario Infanta Leonor, 28031 Madrid, Spain; nmunozr@salud.madrid.org; 9 Service de Médecine Interne, Diabètes et Maladies Métaboliques, Hôpitaux Universitaires de Strasbourg, 67000 Strasbourg, France; noellorenzo@gmail.com

**Keywords:** *Clostridium difficile* infection, heart failure, in-hospital mortality, hospitalization, incidence

## Abstract

Background: We aimed to (1) analyze time trends in the incidence and in-hospital outcomes of heart failure (HF) patients suffering *Clostridioides difficile* infection (CDI); (2) compare clinical characteristics of CDI patients between those with HF and matched non-HF patients; and (3) identify predictors of in-hospital mortality (IHM) among HF patients suffering CDI. Methods: Retrospective study using the Spanish National Hospital Discharge Database from 2001 to 2015. Patients of age ≥40 years with CDI were included. For each HF patient, we selected a year, age, sex, and readmission status-matched non-HF patient. Results: We found 44,695 patients hospitalized with CDI (15.46% with HF). HF patients had a higher incidence of CDI (202.05 vs. 145.09 per 100,000 hospitalizations) than patients without HF (adjusted IRR 1.35; 95% CI 1.31–1.40). IHM was significantly higher in patients with HF when CDI was coded as primary (18.39% vs. 7.63%; *p* < 0.001) and secondary diagnosis (21.12% vs. 14.76%; *p* < 0.001). Among HF patient’s predictor of IHM were older age (OR 8.80; 95% CI 2.55–20.33 for ≥85 years old), those with more comorbidities (OR 1.68; 95% CI 1.12–2.53 for those with Charlson Comorbidity index ≥2), and in those with severe CDI (OR 6.19; 95% CI 3.80–10.02). Conclusions: This research showed that incidence of CDI was higher in HF than non-HF patients. HF is a risk factor for IHM after suffering CDI.

## 1. Introduction

*Clostridioides difficile* infections (CDI) are a common cause of diarrhea in hospitalized patients and studies conducted in several countries have found an increase in the number of cases, as well as in the morbidity and the mortality as a consequence of these infections in the last decades [1,2,3].

In Spain, the National Health System is a public health care insurance system with universal coverage and are fully financed by the general tax fund, so nationwide data are available for infectious diseases such as CDI [4]. The incidence of CDI has increased over time in Spain, with an estimated incidence in 2012 of 17.1 episodes/year/10,000 hospital discharges [4,5]. In 2012, the cost to the National Health System was 32,157,093 €, with a mean cost per episode of the first infection of 3901 € rising to 4875 € for first recurrence and up to 5916 € for a second recurrence [5].

Several reports have analyzed the time trends in the incidence and the CDI-attributable mortality in the last years find conflicting results [6,7,8,9]. In the United States (US), time trends show an increase in mortality from pre-2000 estimates to recent periods [6]. However, other data from Europe and US suggest that the incidence of CDI might have reached a crescendo in the last years and it seems to be reaching a plateau [7]. Wilcox et al. [8] reported that, in England, access to *C. difficile* ribo-typing was followed by a significant reduction in CDI incidence and mortality. These authors suggest that a possible explanation for the decrease in the incidence improvements of control and prevention programs is the variation in the CDI strains over time, or a combination of both factors [7,9].

Risk factors for CDI that have been previously described include, among others; use of antibiotics, elderly populations, prolonged hospital stay, readmissions and comorbid conditions. Previous studies have identified heart failure (HF) as a risk factor associated with CDI [10,11,12].

Using the National Inpatient Sample data for year 2012, Mamic et al. [12] found that patients hospitalized with HF had a 13% greater probability of suffering a CDI (Odds ratio 1.13, 95% CI 1.10–1.16) than patients without HF, after controlling for confounding factors, such as hospital characteristics, concomitant conditions, and patient demographics.

In this investigation, we used the Spanish National Hospital Discharge Database (SNHDD) from 2001 to 2015, and we aim to (i) analyze time trends in the incidence and in-hospital outcomes of HF patients suffering CDI; (ii) compare clinical characteristics of CDI patients between those with HF and matched non-HF patients; and (iii) identify predictors of in-hospital mortality (IHM) among HF patients suffering CDI.

## 2. Materials and Methods

### 2.1. Design, Setting, and Participants

We performed an observational study using data extracted from the SNHDD (Madrid, Spain). Details of the SNHDD characteristics have been published before [13,14,15,16]. For the purposes of this study we selected patients aged 40 and over, hospitalized from 2001 to 2015. We excluded those under 40 because they represent a very small proportion of all hospitalizations for HF in Spain (<1%), and these patients usually have HF as a consequence of congenital disease so are clinically different from the usual HF sufferer [17].

All patients with an International Classification of Diseases, Ninth Revision, Clinical Modification (ICD-9-CM) code for CDI (008.45) in primary or secondary position were selected. According to the SNHDD the primary/main diagnosis is the condition which, after investigation, is considered to be the main reason for admitting the patient to the hospital. The secondary diagnosis includes all those risk factors or diseases that were present at the time of admission or were evoked during hospitalization and that, according to the treating physician, might have affected the patient’s treatment or progress [13]. The ICD-9-CM used in the SNHDD does not include a modifier for diagnoses present on admission [13].

We defined “severe CDI” as those patients who experienced one or more of the following conditions—septicemia, perforation, septic shock, colectomy, perforation or toxic megacolon, in both primary (#1) or secondary diagnosis positions (#2–#14), as described by Gomez-Simmonds et al. [18], using the ICD-9-CM.

Definition of a HF case was based on the recommendations of the American College of Cardiology (ACC)/American Heart Association (AHA) task force on performance measures [19]. We considered HF sufferers’ subjects with any of the following codes in any diagnostic position (ICD-9-CM codes: 398.91, 402.01, 402.11, 402.91, 404.01, 404.03, 404.11, 404.13, 404.91, 404.93, 425.4–425.9, 428.x), while non-heart failure were those without these codes.

The validity of ICD-9 codes for CDI and for HF has not been assessed in the SNHDD database. However, CDI studies conducted in the United States found a good correlation between microbiologic data and ICD-9 codes with sensitivities ranging from 71% to 78% and specificity >99% [20,21,22]. Regarding HF, a review of nineteen studies published from 1999–2009 reporting on the validity of diagnostic codes for identifying HF in administrative data (14 using ICD-9-CM) concluded that although the HF diagnoses identified using administrative data frequently correspond to true HF cases (Specificity was >95% in all studies), this data source might not capture all cases (sensitivity >69% only in 50% of studies) [23].

Once we had the database with heart failure patients with CDI (*n* = 6914), we selected a matched non-HF patient with exactly the same sex, age, year of hospitalization and diagnosis position of CDI (primary or secondary). If more than one control was available for a case, the selection was conducted randomly. When a control is assigned to a case it cannot be used for another case. We identified 1468 and 5327 pairs of patients with a primary and secondary diagnosis of CDI, respectively (98.45% and 98.22% of HF cases matched, respectively).

### 2.2. Main Outcomes Measures

For each patient, the Charlson comorbidity index (CCI) was calculated [24]. The CCI is obtained using diagnoses present on admission and diagnoses appearing during hospital stay. We categorized the CCI in three categories (0, 1, and 2 or more) because it provides well-balanced groups with similar sizes and because this method is commonly used when the CCI is used in epidemiological investigations [14,15,16,17].

Specific risk factors considered in the data analysis included lipid metabolism disorders (ICD-9-CM code 272) recorded during the hospitalization with CDI in any diagnostic position. We included code ICD-9-CM 272 for lipid metabolism disorders because previous studies have suggested that the use of statins might reduce the risk of CDI development or improve the clinical outcomes of CDI [11,25]. Therefore, as the SNHDD does not include medications, we used the ICD-9-CM 272 code as a proxy for statin consumption. We also analyzed parenteral antibiotic therapy (ICD-9-CM 99.21 code) in any procedure position.

Hospitalization variables included admission through the emergency room (ER) length of hospital stay (LOHS), IHM, and readmissions (this variable was included in the database when provided to us by the Ministry of Health).

According to the SNHDD coding, patients can be admitted to the hospital through the ER or with a scheduled admission for a medical treatment or for a diagnosis or surgical procedure. A subject is considered a readmission if the patient had been previously admitted in the same hospital within the last month. Diagnosis-Related Groups (DRG) were used to assess the costs of hospitalization [26,27]. Costs were normalized, considering inflation, to the last year (2015). When the SNHDD is provided to us by the Spanish Ministry of Health, each patient has a specific cost that is calculated by each hospital according to the DRG. The DRG classifies the patients treated in homogeneous groups according to the pathology treated and the consumption of resources. The DRG is also used to identify those patients who have undergone a surgical procedure during the hospitalization. More details in the use of DRG in the SNHDD can be found elsewhere [27].

### 2.3. Statistical Methods

In order to make data easy to read we describe the temporal data using three time periods (2001–2005, 2006–2010, and 2011–2015). The statistical methods have been described before by our group [14,15]. Briefly, descriptive statistics included proportions, means with standard deviations (SD), and medians with interquartile ranges (IQR). A bivariable analysis was done, depending on the variable type, with *t*-test, Mann–Whitney, Chi-square test, ANOVA or Kruskall-Wallis tests. The Bonferroni method was used as a post-hoc test if required.

We used the Poisson regression mode, adjusted by age and sex, to assess changes in incidence, over time (2001–2015).

We constructed bivariate conditional logistic regression models to compare the distribution and the IHM, according to study variables between patients with HF and matched controls.

Predictors of IHM among HF patients according to the CDI diagnosis position were identified using unconditional logistic regression analyses.

The multivariable models were built using the “enter modelling” method of STATA 14.0. The process included the following steps:

(1) Each independent variable was analyzed according to the diagnosis position of CDI (primary, secondary, both), (bivariate analysis).
(2) Selection of variables for the multivariable analysis. We included those significant variables in the bivariate analysis and those found scientifically relevant by other authors.
(3) To fit the multivariable model we used the Wald statistic (WS) to assess the importance of each variable. The variables that did not improve the model according to the tests we considered, were deleted, and a new model was re-analyzed (expert opinion method for independent variable selection). Likelihood Ratio test was used to compare successive models with previous ones.
(4) Once the final model was fitted, we checked for collinearity and interactions between the remaining variables.

Stata version 14 (Stata, College Station, TX, USA) was used for statistical analyses and matching. Statistical significance was set at *p* < 0.05 (2-tailed).

### 2.4. Ethical Aspects

The SNHDD is owned by the Spanish Ministry of Health, who provided us the database. When we received the database, all personal identifiers were deleted, therefore, confidentiality was fully warrantied. According to the Spanish legislation, given the type of data used in our investigation, it is not necessary to present and obtain the approval by an ethics committee.

## 3. Results

We identified 44,695 hospitalizations of patients aged ≥40 years suffering CDI from 2001 to 2015. A total of 15.46% of patients with CDI had HF codified in their discharge report; 4175 were women and 2739 were men.

### 3.1. Incidence, Clinical Variables, and In-Hospital Characteristics of Patients with Heart Failure Who Suffered Clostridium Difficile Infection

Table 1 shows the trends in the incidence, the clinical variables, and in-hospital characteristics of patients with HF who suffered a CDI.

Among patients with HF, the incidence of CDI rose from 143.34 cases per 100,000 hospitalizations with HF in 2001–2005 to 256.66 in 2011–2015 (*p* < 0.001). As can be seen in Table 1, the total incidence of CDI from 2001 to 2015 among HF sufferers was higher than that among non-HF sufferers (202.05 vs. 145.09 per 100,000 hospitalizations; *p* < 0.001). These incidences were also significantly higher among HF sufferers for each single time period analyzed.

Results of the age and sex-adjusted Poisson regression models yielded an adjusted IRR of 1.35 (95% CI, 1.31–1.40). Therefore, compared with the previous time period, the incidence of hospitalization increased by mean a 35% in every time period.

Age increased significantly over time (80.27, SD 9.61 years in 2001–2005 vs. 81.18, SD 9.53 years in 2011–2015; *p* = 0.018) as well as the proportion with hypercholesterolemia (8.67% vs. 20.59%; *p* < 0.001) and parenteral antibiotic therapy (23.29% vs. 41.81%). On the other hand, the proportion of patients that underwent a surgical procedure decreased over time by 3% from 15.36% to 11.34%.

The presence of CDI as the primary diagnosis increased significantly (12.47% in 2001–2005 vs. 25.95% in 2011–2015; *p* < 0.001) as did the severe CDI cases (5.62% vs. 11.72%; *p* < 0.001). Distribution according to the female sex and CCI showed no significant change over time.

Median LOHS for admissions for CDI was 26 days (IQR 26) in the period 2001–2005, decreasing to 18 days (IQR 19) in 2011–2015. In contrast, the mean cost per patient rose from 4300.85 € (SD 2329.68) in the period 2001–2005 to 4476.63 € (SD 1940.14) in 2011–2015. The proportion of ER admissions and readmissions also increased from 86.79% to 24.11%, respectively, in the period 2001–2005 to 90.15% and 36.81%, respectively, in 2011–2015 (*p* < 0.001). For the total time period, crude IHM among CDI cases was 20.68% and decreased significantly from 21.88% in 2001–2005 to 19.42% in 2011–2015 (*p* = 0.022).

### 3.2. Clinical Characteristics and In-Hospital Outcomes in Patients with Heart Failure According to Primary and Secondary Clostridium Difficile Infection Diagnosis

Table 2 shows data regarding clinical characteristics and in-hospital outcomes in patients with HF, according to primary and secondary CDI diagnosis. The proportion of hospitalizations with CDI as the primary diagnosis increased from 21.66% in 2001–2005 to 47.46% in 2011–2015 (*p* < 0.001). Females were significantly more represented among patients with a primary diagnosis of CDI (62.63% vs. 59.76%; *p* = 0.045). Patients with CDI as the primary diagnosis were significantly older (81.76; SD = 8.85 years) than patients with CDI as the secondary diagnosis (80.75; SD = 9.66 years), had more coexisting medical conditions (CCI ≥2: 41.22% vs. 38.22%; *p* = 0.037), and had more ER admission (91.64% vs. 89.52%; *p* = 0.015). However, the use of parenteral antibiotic therapy, surgical procedures, severity of CDI, readmission, and mean LOHS was significantly higher in patients with CDI as the secondary diagnosis. Furthermore, IHM was higher in patients with a secondary diagnosis of CDI (21.26% vs. 18.58%; *p* = 0.024).

### 3.3. Distribution According to the Study Variables of Heart Failure Patients and Matched Non-Heart Failure Controls with Diagnosis of Clostridium Difficile Infection

Table 3 shows the distribution according to the study variables of HF patients and matched non-HF controls with a diagnosis of CDI. The number of hospitalizations of patients with HF increased from 1180 (17.37%) in 2001–2005 to 3636 (53.51%) in the last period analyzed (*p* < 0.001). A total of 21.6% patients with HF had CDI as the primary diagnosis and 60.31% were women. The mean age was 80.75 years.

After matching, patients with HF had significantly more comorbidity (mean CCI, 1.32, SD 0.99 vs. 1.2, SD 0.97, *p* < 0.001) than non-HF patients. The ER admission and readmission rates were significantly higher in HF patients than in those without HF (90.15% and 32.8% vs. 88.34% and 29.93%, respectively; all *p* < 0.001). Median LOHS was higher in HF patients (20 days IQR 22 vs. 17 days IQR 20; *p* < 0.001).

As shown in the Supplementary Table S1, we found that overall IHM was significantly higher in patients with HF than in the matched non-HF controls, when CDI was coded as primary (18.39% vs. 7.63% *p* < 0.001) or secondary diagnosis (21.12% vs. 14.76%, *p* < 0.001). IHM was significantly higher in all age groups, controlling for sex, among patients with HF than among matched non-HF controls.

Patients with HF who had hypercholesterolemia and a higher severity of CDI had higher values of IHM than control patients (13.69% and 40.87% vs. 8.17% and 32.24%; all *p* < 0.001). Furthermore, patients with HF admitted through the ER and who were readmitted had higher IHM than their matched non-HF controls (19.98% and 22.75% vs. 12.86% and 14.45%, respectively, with *p*-values of <0.001 and 0.004).

The IHM in patients with HF as primary diagnosis and severe CDI was 54.55%, compared to 17.49% for those without severe CDI (*p* < 0.001). The corresponding figures for those with HF as a secondary diagnosis were 40.21% and 18.66%, respectively (*p* < 0.001).

Finally, in the bivariate analysis all HF patients (primary or secondary position) with severe CDI had 8.63% (40.87% vs. 32.24%) higher IHM than matched non-HF patients.

### 3.4. Predictors of In-Hospital Mortality in Heart Failure Patients with Clostridium Difficile Infection

Table 4 shows the predictors of IHM in HF patients with CDI codified in primary, secondary, and any diagnosis position. Only variables with a significant OR are shown in the table.

When CDI appeared as the primary diagnosis, IHM was significantly higher in older subjects (OR 8.80, 95% CI 2.55–20.33 for ≥85 years old vs. <40–64 years old), higher values in the CCI (OR 1.68, 95% CI 1.12–2.53 vs. no comorbidities) and in those considered to have severe CDI (OR 6.19, 95% CI 3.80–10.02).

Predictor of IHM when CDI is a secondary diagnosis are the same as when recorded in a primary position. The time trend analysis showed a significant decrease in IHM over time, only in patients with a secondary diagnosis of CDI. Variables associated with a lower risk of dying among HF patients with a secondary diagnosis of CDI included, being female (OR 0.85; 95% CI 0.75–0.99), a diagnosis of hypercholesterolemia (OR 0.59; 95% CI 0.47–0.73), and admission through the ER (OR 0.71; 95% CI 0.58–0.87).

The diagnosis position of CDI among HF patients was not associated with IHM after adjustment for possible confounders (OR, 0.93; 95% CI 0.79–1.08).

## 4. Discussion

The main results of our investigation are that patients with HF have higher incidence rates of hospitalization for CDI than patients without HF. A study carried out in the United States agrees with us and demonstrated that patients with HF had higher rates of CDI [12].

In our study, the incidence of CDI among HF patients increased over time. This increase could be due to factors that affect the general population, such as an increase in antibiotic prescriptions, comorbidities, greater use of diagnostic test for CDI, and older age of hospitalized patients [11,25,28,29]. In the specific case of HF, it should be noted that the splenic circulation congestion might induce some changes in the intestinal microbiota, leading to the development of CDI during hospitalization [30]. In addition, patients with HF are more likely to be malnourished and this condition might also lead to CDI infections and its complications, observed over time [12,30,31].

The severe cases observed could be a consequence of the patient’s clinical situation itself (HF; malnutrition; antibiotic treatment received; prolonged hospital stay) or the result of infection by strains resistant to metronidazole and vancomycin [30,31,32].

Notably, IHM due to CDI in patients with HF decreased during the study period. This could probably be the result of the use of new therapies, such as fidaxomicin or fecal transplantation, although we have no data to confirm this hypothesis [32].

An interesting result of our investigation is that, while the number of, both, overall and severe CDI diagnoses increased over the study period, in-hospital mortality and length of stay both decreased. This suggests that over-coding or increased diagnoses might be responsible for the observed increase [33,34]. In the US, Cohen et al. suggested that a potential reason for decreased *C. difficile*-associated fatality could be the mislabeling of *C. difficile* carriers as *C. difficile* infection cases [33]. Their report describes a survey-based study of 121 laboratories participating in the CDC Emerging Infections Program population-based *C. difficile* infection surveillance. In these laboratories the use of PCR testing for *C. difficile* diagnosis had increased over time, with 43% of laboratories using this testing in 2011. This shift by laboratories toward PCR has led to a 45% increase in positive results, raising concerns about the detection of colonized patients [33]. Polage et al. [34] have found that the exclusive reliance on PCR for CDI is likely to result in some grade of over-diagnosis and over-treatment.

Future investigations should analyze the use of PCR in Spanish hospital laboratories, in order to assess a possible over diagnosis of CDI that could result in a delay in the detection and treatment of alternative causes of diarrhea. It will also be of interest for future prospective studies to determine which phenotype of HF patients are prone to developing CDI or have a CDI colonization. Recent studies have shown that patients with the right HF are prone to develop intestinal congestion, malabsorption, and bacterial translocation, as described in liver cirrhosis [30,31,35].

In Spain, specific CDI surveillance systems should be implemented in hospitals, including detailed microbiological data, in order to identify strains and microbiological susceptibility, as well as information on adverse outcomes [14,15,16]. To do so, the use of standardized protocols such as the European Surveillance Protocol provided by the European Centre for Disease Prevention and Control is recommended [36].

Interestingly, it is unclear if patients with HF have higher rates of CDI as the result of higher infection rates and subsequently antibiotic treatment or if heart failure itself increases susceptibility to CDI when antibiotics are prescribed. Many factors could be associated with the increase risk of CDI in this group of patients—(a) higher rates of hospitalizations and subsequently exposure to CD, (b) higher rates of infections and antibiotic use, (c) gut structure changes and microbiota disruption, and (d) higher density of bacteria in the sigmoid mucosal biofilm and active bacteria proliferation [11,25,28,29].

Patient with HF have been considered to be more sensitive to the inflammatory response during CDI. The changes in the gut mucosa constitute an important step in the role of toxins stimulating cytokines production and catecholamine release. This inflammatory response could have a pejorative effect in the progression of heart failure. Treatment against asymptomatic CD carriers is not currently recommended. However, this might be considered if CDI is shown to increase mortality and morbidity in heart failure. In this setting, prophylactic measures such as probiotics and prebiotics might also be considered [12,30,31].

Some limitations should be considered. First, the SNHDD, is an administrative database and is not designed for epidemiological investigation. As any discharge database, it is possible that errors in coding exist and changes in the coding practices over time might occur. Additionally, the definition of HF could have been modified along the study period. Given the characteristics of the SNHDD, we missed data on pharmacological treatment or specific clinical issues of CDI. Our findings are also limited by the lack of data on left ventricular ejection fraction measurement and NT-proBNP levels, to evaluate the severity of HF during admissions. Second, no specific code is available to differentiate community-onset or nosocomial-acquired or CDI.

Third, unfortunately, the SNHDD uses the ICD9CM and this coding system does not include a “present on admission (POA)” indicator code. So it is not possible to know if HF was a consequence of another condition such as pneumonia, acute cardiac ischemia, or kidney failure, among others. Despite these limitations, we provide data for an entire country for 15 years, which has previously been proved useful to assess CDI in Spain [14,15,16].

## 5. Conclusions

Patients with HF have higher incidence rates of hospitalization for CDI than in patients without HF and the incidence is increasing over time. IHM was significantly higher in all age groups, after controlling for sex, among patients with HF than matched non-HF controls. This higher mortality was found for CDI when coded as a primary or secondary diagnosis.

An interesting result of our investigation is that while the number of overall and severe CDI diagnoses increased over the study period, in-hospital mortality and length of stay both decreased. This suggests that over-coding or increased diagnoses might be responsible for the observed increase and make necessary further investigations to clarify this point.

## Figures and Tables

**Table 1:** Characteristics of hospital admissions with *Clostridioides difficile* infection (CDI) among patients suffering heart failure (HF) in Spain, from 2001 to 2015, and incidence among non HF patients.

Variable	Categories	2001–2005	2006–2010	2011–2015	Total
Total number of hospital admissions with HF		844,845	1,144,810	1,432,245	3,421,900
Number of hospital admissions with CDI in HF patients		1211	2027	3676	6914
Incidence per 100,000 HF admissions *		143.39	177.06	256.66	202.05
Number of hospital admissions with CDI without HF		6921	11,282	19,578	37,781
Incidence per 100,000 non-HF admissions *		104.76	120.41	182.21	145.09
CDI as a primary diagnosis. *N* (%) *		151(12.47)	386(19.04)	954(25.95)	1491(21.56)
Female sex. *N* (%)		724(59.79)	1222(60.29)	2229(60.64)	4175(60.38)
Age in years. Mean (SD) *		80.27(9.61)	81.14(9.36)	81.18(9.53)	80.97(9.5)
Age groups in years *	40–64	82(6.77)	115(5.67)	240(6.53)	437(6.32)
	65–74	201(16.6)	308(15.19)	473(12.87)	982(14.2)
	75–84	479(39.55)	794(39.17)	1491(40.56)	2764(39.98)
	≥85	449(37.08)	810(39.96)	1472(40.04)	2731(39.5)
CCI. Mean (SD)		1.31(1.01)	1.28(0.98)	1.33(0.99)	1.31(0.99)
CCI. *N* (%)	0	266(21.97)	451(22.25)	769(20.92)	1486(21.49)
	1	472(38.98)	807(39.81)	1461(39.74)	2740(39.63)
	≥2	473(39.06)	769(37.94)	1446(39.34)	2688(38.88)
Hypercholesterolemia. *N* (%) *	Yes	105(8.67)	234(11.54)	757(20.59)	1096(15.85)
Parenteral antibiotic therapy. *N* (%) *	Yes	282(23.29)	658(32.46)	1537(41.81)	2477(35.83)
Surgery *N* (%) *	Yes	186(15.36)	244(12.04)	417(11.34)	847(12.25)
Severity. *N* (%) *	Yes	68(5.62)	199(9.82)	431(11.72)	698(10.1)
Readmission. *N* (%) *	Yes	292(24.11)	617(30.44)	1353(36.81)	2262(32.72)
ER admission. *N* (%) *	Yes	1051(86.79)	1856(91.56)	3314(90.15)	6221(89.98)
IHM. *N* (%) *		265(21.88)	451(22.25)	714(19.42)	1430(20.68)
LOHS. Median (IQR) *		26(26)	22(24)	18(19)	20(22)
Cost. Mean (SD) *		4300.85(2329.68)	4737.32(2565.73)	4476.63(1940.14)	4508.08(1951.52)

HF—Heart Failure. CDI— *Clostridioides difficile* infection. CCI—Charlson Comorbidity Index. ER—Emergency room. LOHS—Length of hospital stay. IHM—In-hospital mortality. SD—Standard Deviation. IQR—Interquartile range. * *p* < 0.05 to assess time trend from 2001 to 2015.

**Table 2:** Characteristics of hospital admissions of patients with primary diagnosis and patients with secondary diagnosis of *Clostridioides difficile* infection among patients suffering heart failure in Spain, from 2001 to 2015.

Variable	Categories	Primary Diagnosis	Secondary Diagnosis	*p*
Year. *N* (%)	2001–2005	324(21.66)	887(16.37)	<0.001
	2006–2010	462(30.88)	1565(28.89)	
	2011–2015	710(47.46)	2966(54.74)	
Sex. *N* (%)	Male	559(37.37)	2180(40.24)	0.045
	Female	937(62.63)	3238(59.76)	
Age in years. Mean (SD)		81.76(8.85)	80.75(9.66)	<0.001
Age groups in years	40–64	74(4.95)	363(6.7)	0.004
	65–74	191(12.77)	791(14.6)	
	75–84	594(39.71)	2170(40.05)	
	≥85	637(42.58)	2094(38.65)	
CCI. Mean (SD)		1.37(0.97)	1.3(0.99)	0.020
CCI. *N* (%)	0	281(18.78)	1205(22.24)	0.037
	1	598(39.97)	2142(39.53)	
	≥2	617(41.24)	2071(38.22)	
Hypercholesterolemia. *N* (%)	Yes	260(17.38)	836(15.43)	0.068
Parenteral antibiotic therapy *N* (%)	Yes	466 (31.25)	2011(37.08)	<0.001
Surgery *N* (%)	Yes	28(1.88)	819(15.10)	<0.001
Severity. *N* (%)	Yes	44(2.94)	654(12.07)	<0.001
Readmission *N* (%)	Yes	456(30.48)	1806(33.33)	0.037
ER admission. *N* (%)	Yes	1371(91.64)	4850(89.52)	0.015
IHM. *N* (%)		278(18.58)	1152(21.26)	0.024
LOHS. Median (IQR)		20(18)	21(23)	<0.001
Cost. Mean (SD)		4584.12(1844.04)	4508.08(2622.84)	0.059

*p*-value for the difference between patients with primary diagnosis of CDI and patients with secondary diagnosis. CCI—Charlson Comorbidity Index. ER—Emergency room. LOHS—Length of hospital stay. IHM—In-hospital mortality. SD—Standard Deviation. IQR—Interquartile range.

**Table 3:** Distribution of heart failure (HF) patients and matched non-HF controls with a diagnosis of *Clostridioides difficile* infection according to the variables studied.

Variable	Categories	HF	Matched Non-HF	*p*
Diagnosis position	Primary	1468(21.6)	1468(21.6)	NA
	Secondary	5327(78.4)	5327(78.4)	
Year *N* (%)	2001–2005	1180(17.37)	1180(17.37)	NA
	2006–2010	1979(29.12)	1979(29.12)	
	2011–2015	3636(53.51)	3636(53.51)	
Sex. *N* (%)	Female	4098(60.31)	4098(60.31)	NA
	Male	2697(39.69)	2697(39.69)	
Age in years. Mean (SD)		80.75(9.37)	80.75(9.37)	NA
Age groups in years. Mean (SD)	40–64	434(6.39)	434(6.39)	NA
	65–74	980(14.42)	980(14.42)	
	75–84	2764(40.68)	2764(40.68)	
	≥85	2617(38.51)	2617(38.51)	
CCI. Mean (SD)		1.32(0.99)	1.2(0.97)	<0.001
CCI. *N* (%)	0	1446(21.28)	1737(25.56)	<0.001
	1	2697(39.69)	2756(40.56)	
	≥2	2652(39.03)	2302(33.88)	
Hypercholesterolemia. *N* (%)	Yes	1088(16.01)	1212(17.84)	0.005
Parenteral antibiotic therapy *N* (%)	Yes	2432(35.79)	2358(34.70)	0.181
Surgery *N* (%)	Yes	842(12.39)	900(13.24)	<0.191
Severity. *N* (%)	Yes	690(10.15)	729(10.73)	0.274
Readmission *N* (%)	Yes	2229(32.8)	2034(29.93)	<0.001
ER admission. *N* (%)	Yes	6126(90.15)	6003(88.34)	<0.001
LOHS. Median (IQR)	Yes	20(22)	17(20)	<0.001
Cost. Mean (SD)		4508.08(1944.5)	4422.41(2846.64)	0.563

The *p*-value for the difference between patients with HF and matched controls was calculated with the bivariate conditional logistic regression model. CCI—Charlson Comorbidity Index. ER—Emergency room. LOHS—Length of hospital stay. IHM—In-hospital mortality. SD—Standard Deviation. IQR—Interquartile range. NA—Not applicable as it is a matching variable.

**Table 4:** Multivariable analysis of factors associated with in-hospital mortality among HF patients with *Clostridioides difficile* infection (CDI) according to the diagnosis position.

Variable	Categories	Primary Diagnosis		Secondary Diagnosis		Any Position	
		Crude OR (95% CI)	Adjusted OR (95% CI)	Crude OR (95% CI)	Adjusted OR (95% CI)	Crude OR (95% CI)	Adjusted OR (95% CI)
Year	2001–2005	1	1	1	1	1	1
	2006–2010	1.22(0.77–1.96)	1.14(0.70–1.85)	0.99(0.82–1.19)	0.93(0.76–1.12)	1.02(0.86–1.21)	0.96(0.81–1.15)
	2011–2015	0.78(0.51–1.21)	0.76(0.48–1.20)	0.91(0.76–1.08)	0.83(0.70–0.99)	0.86(0.73–1.00)	0.82(0.69–0.96)
Sex	Female	0.77(0.59–1.01)	0.76(0.57–1.02)	0.91(0.80–1.04)	0.85(0.75–0.99)	0.87(0.77–0.98)	0.84(0.73–0.95)
Age groups in years	40–64	1	1	1	1	1	1
	65–74	4.19(1.23–14.23)	5.69(1.59–20.42)	1.27(0.91–1.77)	1.39(0.99–1.96)	1.41(1.04–1.93)	1.58(1.14–2.18)
	75–84	3.93(1.21–12.77)	6.13(1.78–21.10)	1.39(1.03–1.88)	1.66(1.22–2.27)	1.50(1.12–1.99)	1.85(1.37–2.48)
	≥85	5.06(1.56–16.44)	8.80(2.55–30.33)	1.78(1.32–2.39)	2.28(1.67–3.11)	1.91(1.44–2.54)	2.55(1.89–3.43)
CCI	0	1	1	1	1	1	1
	1	1.54(1.06–2.24)	1.89(1.27–2.83)	1.19(1.00–1.42)	1.33(1.11–1.59)	1.25(1.07–1.47)	1.41(1.19–1.66)
	≥2	1.34(0.92–1.94)	1.68(1.12–2.53)	1.07(0.89–1.28)	1.25(1.04–1.52)	1.11(0.95–1.31)	1.32(1.11–1.56)
Hypercholesterolemia	Yes	0.64(0.45–0.91)	0.72(0.50–1.04)	0.55(0.44–0.68)	0.59(0.47–0.73)	0.57(0.47–0.68)	0.62(0.51–0.75)
Severity	Yes	5.46(3.49–8.54)	6.19(3.80–10.02)	2.80(2.35–3.35)	3.06(2.55–3.69)	3.09(2.63–3.65)	3.37(2.84–4.00)
Readmission	Yes	1.07(0.82–1.39)	1.09(0.82–1.43)	1.31(1.14–1.51)	1.42(1.23–1.64)	1.21(1.07–1.37)	1.33(1.18–1.52)
ER admission	Yes	0.67(0.41–1.11)	0.70(0.42–1.18)	0.74(0.60–0.89)	0.71(0.58–0.87)	0.72(0.60–0.86)	0.71(0.59–0.86)
Primary diagnosis	Yes	NA	NA	NA	NA	0.83(0.72–0.97)	0.93(0.79–1.08)

CCI—Charlson Comorbidity Index. ER—Emergency room. OR—Odds ratio obtained using logistic regression models. 95% CI; 95% confidence intervals. Only those variable that showed a significant association are showed. NA—Not applicable.

## Supplementary Materials

The following are available online at https://www.mdpi.com/2077-0383/9/3/614/s1, Table S1: In-hospital mortality according to study variables of heart failure (HF) patients and matched non-HF controls with a diagnosis *of Clostridioides difficile* infection.
