# Supplementary material for: Heart Failure Is a Risk Factor for Suffering and Dying of Clostridium difficile Infection. Results of a 15-Year Nationwide Study in Spain
Source: J Clin Med. 2020 Feb 25;9(3):614. doi: 10.3390/jcm9030614 (PMC7141109; doi:10.3390/jcm9030614)
Supplement: Supplementary file 1 [file jcm-09-00614-s001.pdf]

Supplementary Table 1. In-hospital mortality according to study variables of heart failure (HF) patients and matched non-HF controls with a diagnosis *of Clostridioides difficile* infection.

		**IHM HF**	**IHM Matched non HF**	***p***
Diagnosis position	Primary	270(18.39)	112(7.63)	<0.001
	Secondary	1125(21.12)	786(14.76)	<0.001
Year *N* (%)	2001-05	256(21.69)	194(16.44)	0.001
	2006-10	437(22.08)	275(13.9)	<0.001
	2011-15	702(19.31)	429(11.8)	<0.001
Sex. *N* (%)	Female	802(19.57)	526(12.84)	<0.001
	Male	593(21.99)	372(13.79)	<0.001
Age in years. Mean (SD)		81.91(8.88)	82.3(8.73)	0.356
Age groups in years.	40-64	59(13.59)	38(8.76)	0.024
Mean (SD)	65-74	183(18.67)	101(10.31)	<0.001
	75-84	540(19.54)	350(12.66)	<0.001
	≥85	613(23.42)	409(15.63)	<0.001
CCI. Mean (SD)		1.35(0.99)	1.3(1.01)	0.293
CCI. *N* (%)	0	265(18.33)	197(11.34)	0.014
	1	596(22.1)	374(13.57)	<0.001
	≥2	534(20.14)	327(14.21)	0.015
Hypercholesterolemia. *N* (%)	Yes	149(13.69)	99(8.17)	<0.001
Parenteral antibiotic therapy *N* (%)	Yes	467(19.20)	425(18.02)	0.385
Surgery *N* (%)	Yes	180(21.38)	177(19.67)	0.472
Severity. *N* (%)	Yes	282(40.87)	235(32.24)	<0.001
Readmission *N* (%)	Yes	507(22.75)	294(14.45)	0.004
ER admission. *N* (%)	Yes	1224(19.98)	772(12.86)	<0.001
LOHS. Median (IQR)	Yes	23(25)	22(26)	0.209
Cost. Mean (SD)		4740.4 (2244.77)	4994.67 (3135.4)	0.494

The *p* value for the difference between patients with HF patients and matched controls was calculated with the bivariate conditional logistic regression model. CCI Charlson Comorbidity Index. ER Emergency room. LOHS Length of hospital stay. IHM In hospital mortality. SD Standard Deviation. IQR. Interquartile range. NA. Not applicable as this is a matching variable
